# The TNM 8^th^ edition: Validation of the proposal for organ - confined (pT2) prostate cancer

**DOI:** 10.1590/S1677-5538.IBJU.2018.0338

**Published:** 2019-04-01

**Authors:** Athanase Billis, Leandro L. L. Freitas, Larissa B. E. Costa, Icleia S. Barreto, Luis A. Magna, Wagner E. Matheus, Ubirajara Ferreira

**Affiliations:** 1 Departamento de Anatomia Patológica, Faculdade de Ciências Médicas, Universidade Estadual de Campinas (Unicamp), Campinas, SP, Brasil; 2 Departamento de Genética Médica / Bioestatística da Faculdade de Ciências Médicas (Unicamp), Campinas, SP, Brasil; 3 Departmento de Urologia da Faculdade de Ciências Médicas da Universidade Estadual de Campinas (Unicamp), Campinas, SP, Brasil

**Keywords:** Prostate, Prostatic Neoplasms, Pathology, Surgical

## Abstract

**Purpose:**

The 8^th^ edition of the TNM has been updated and improved in order to ensure a high degree of clinical relevance. A major change in prostate includes pathologically organ - confined disease to be considered pT2 and no longer subclassified by extent of involvement or laterality. The aim of this study was to validate this major change.

**Materials and Methods:**

Prostates were step - sectioned from 196 patients submitted to radical prostatectomy with organ confined disease (pT2) and negative surgical margins. Tumor extent was evaluated by a semiquantitative point count method. The dominant nodule extent was recorded as the maximal number of positive points of the largest single focus of cancer from the quadrants. Laterality was considered as either total tumor extent (Group 1) or index tumor extent (Group 2). Time to biochemical recurrence was analyzed with the Kaplan - Meier product limit analysis and prediction of shorter time to biochemical recurrence with Cox proportional hazards model.

**Results:**

In Group 1, 43 / 196 (21.9%) tumors were unilateral and 153 / 196 (78.1%) bilateral and in Group 2, 156 / 196 (79.6%) tumors were unilateral and 40 / 196 (20.4%) bilateral. In both groups, comparing unilateral vs bilateral tumors, there was no significant clinicopathological difference, and no significant association with time as well as prediction of shorter time to biochemical recurrence following surgery.

**Conclusions:**

Pathologic sub - staging of organ confined disease does not convey prognostic information either considering laterality as total tumor extent or index tumor extent. Furthermore, no correlation exists between digital rectal examination and pathologic stage.

## INTRODUCTION

The 8^th^ edition of the TNM has been updated and improved in order to ensure a high degree of clinical relevance (1, 2). Major changes in prostate include: 1) Pathologically organ - confined disease is now considered pT2 and is no longer subclassified by extent of involvement or laterality; 2) Tumor grading now includes both the Gleason score (as in the seventh edition criteria) and the grade group (introduced in the eighth edition criteria); 3) Prognostic stage group III includes select, organ - confined disease based on prostate - specific antigen and Gleason / grade group status; and, 4) Two statistical prediction models are included in the staging manual.

The aim of this study was to validate the major change 1 of the 8^th^ edition of the TNM. We evaluated the tumor in either its total extent or exclusively the index tumor extent. To the best of our knowledge there is no other study considering index tumor extent in sub - staging. Furthermore, we studied the possible correlation between digital rectal examination and pathological stage.

## MATERIALS AND METHODS

This retrospective study was based on 196 consecutive patients in a time period from 1997 to 2015 with organ confined prostate cancer (pT2) and negative surgical margins treated with retropubic RP by 1 surgeon (UF). We compared the biochemical recurrence following surgery of unilateral (pT2a / pT2b) vs bilateral (pT2c) tumors considering either total tumor extent (Group-1) or index tumor extent (Group-2). Several clinicopathological variables were also studied.

After RP, serum PSA was drawn every 3 months during the first year, every 6 months during the second year, and annually thereafter. No patient of this series had radiotherapy or androgen manipulation before or after surgery. Total serum PSA was measured utilizing previous validated Immulite^®^ PSA kit. Biochemical recurrence following surgery was considered as PSA ≥ 0.2ng / mL according to recommendation of the American Urological Association (3). Patients without evidence of BCR were censored at last follow-up. PSA density was calculated using the pathological weight of the prostate without the seminal vesicles. The present study was approved by the Institutional Committee of Ethics of our Institution.

Surgical specimens were step sectioned at 3 to 5 mm intervals and embedded in paraffin. A mean of 32 paraffin blocks was processed. Sections (6 µm) of each block were stained with hematoxylin and eosin. Each transverse section of the prostate was subdivided into 2 anterolateral and 2 posterolateral quadrants. Using the cone method 8 sections from the bladder neck and 8 from the apex were obtained. Each seminal vesicle was sampled with 3 transverse sections: proximal, median, and distal.

Positive surgical margin was defined as cancer cells in contact with the inked specimen surface. Extra - prostatic extension was diagnosed whenever cancer was seen in adipose tissue and, in case of desmoplastic response, when a protuberance corresponding to extension of tumor into peri - prostatic tissue was seen. Seminal vesicle invasion occurred when there was involvement of the muscular coat. RP Gleason grading was stablished according to the 2014 International Society of Urological Pathology (ISUP) consensus conference (4) considering the entire tumor. According to Gleason score the tumors were classified as grade group 1 (≤ 6), grade group 2 (3 + 4 = 7), grade group 3 (4 + 3 = 7), grade group 4 (8), and grade group 5 (9-10). Nodular hyperplasia was considered whenever this pathological finding was in the surgical specimen. Digital rectal examination was recorded as T1c, cT2a / cT2b, and cT2c.

**Figure 1:**
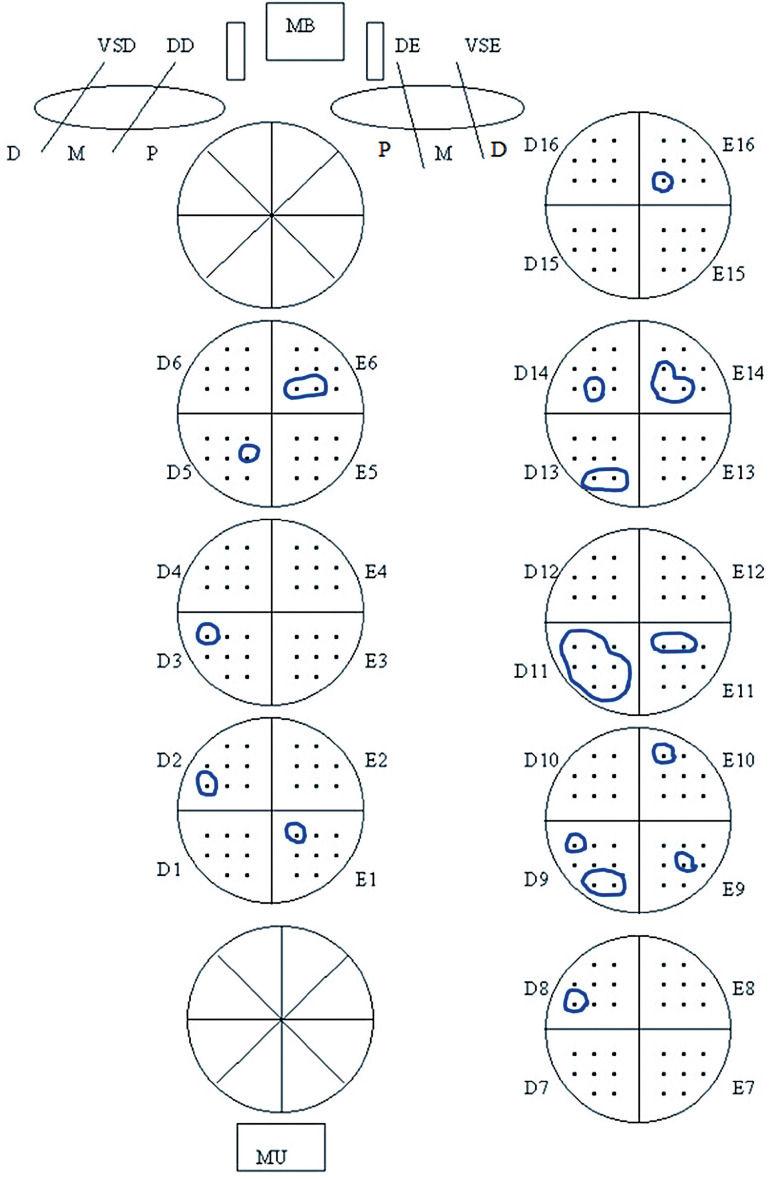
Semiquantitative point count method to evaluate tumor extent. In this case, total tumor extent was recorded as 28 positive points. Quadrant D11 shows largest single cancer focus or dominant nodule of all quadrants, recorded as 7 index tumor positive points. The tumor is bilateral considering total tumor extent, and unilateral considering index tumor extent.

Tumor extent at RP was evaluated by a previously described semiquantitative point count method (5). Briefly, each quadrant of the transverse sections was drawn on paper and contained 8 equidistant points. During microscopic examination of the slides, the tumor area was drawn on the correspondent quadrant on the paper (Figure-1). At the end of examination, the number of positive points represented an estimate of tumor extent but not volume. Each positive point corresponds to 10 – 15% of extent in each quadrant.

Total tumor extent was recorded as the total sum of positive points of all transverse quadrants. Index tumor extent (dominant nodule) was recorded as the maximum number of positive points for the largest single focus of cancer present in the quadrants and not with the highest grade. In Figure-1, total and index tumor extent was recorded as 28 and as 7 positive points, respectively; the tumor is bilateral considering total tumor extent, and unilateral considering index tumor extent. In Group-2, bilateral tumors were considered when index tumor had the same number of positive points on both sides either in adjacent quadrants or not. Even in non - adjacent quadrants, most probably the nodules are not co - dominant. The dominant tumor nodules are rarely symmetrical and mostly will be on one side and part of the nodule will cross the midline. All cases were reviewed by a senior uropathologist (AB).

### Statistical analysis

The data were analyzed using the Qui - square test and Fisher's exact test to compare proportions, the Mann - Whitney test to compare means, Kaplan - Meier product limit analysis for TBCR using the log rank test for comparison between the groups according to laterality, and Cox stepwise logistic regression model was used to identify significant predictors of shorter TBCR. Statistical significance was considered at p < 0.05. All statistical analyses were performed using PASW^®^ Statistics 18.0.

## RESULTS

### Group 1 (total tumor extent)

In this group, 43 / 196 (21.9%) tumors were unilateral and 153 / 196 (78.1%) bilateral.

### Clinicopathological Findings

Except for RP Gleason grade on the limit of significance, there was no significant association comparing bilateral vs unilateral tumors (Table-1).

**Table 1:** Clinicopathological features of 43/196 (21.9%) unilateral tumors vs 153/196 (78.1%) bilateral tumors by total tumor extent.

Feature		Unilateral tumors	Bilateral tumors	p Value
Mean ± SD age/median (range)		61.95 ± 6.63/62 (46-73)	63.05 ± 6.45/64 (46-76)	0.384 (Mann-Whitney test)
Mean ± SD prostate weight/median (range)		41.47 ± 19.52/38 (15-110)	43.08 ± 24.95/36 (15-185)	0.934 (Mann-Whitney test)
**No. nodular hyperplasia (%)**				
	Neg	9 (20.9)	42 (27.8)	0.435 (Fisher exact test)
	Pos	34 (79.1)	109 (78.2)	
Mean ± SD preop PSA/median (range)		8.45 ± 4.60/7.70 (2-20)	7.79 ± 5.12/6.48 (0.60-33)	0.209 (Mann-Whitney test)
Mean ± SD PSA density/median (range)		0.23 ± 0.16/0.20 (0.04-0.85)	0.33 ± 1.56/0.17 (0.03-19.25)	0.135 (Mann-Whitney test)
**No. Grade group (%)**				
	1 (≤6)	29 (67.4)	69 (45.1)	0.057 (Qui-square test)
	2 (3+4=7)	10 (23.3)	70 (45.8)	
	3 (4+3=7)	3 (7.0)	11 (7.2)	
	4 (8)	0 (0.0)	1 (0.7)	
	5 (9-10)	1 (2.3)	2 (1.3)	
**No. clinical stage (%)**				
	T1c	25 (58.1)	82 (56.9)	0.543 (Qui-square test)
	T2a/T2b	18 (41.9)	58 (40.3)	
	T2c	0 (0.0)	4 (2.8)	

### Pathological stage vs. clinical stage by DRE

Only 4 (2.8%) patients were considered cT2c by DRE vs. 153 (78.1%) patients pT2c; and, 18 (41.9%) patients were considered cT2a / cT2b by DRE vs. 43 (21.9%) pT2a / pT2b. Information for clinical stage was missing in 9 patients.

### Time to BCR

**Figure 2:**
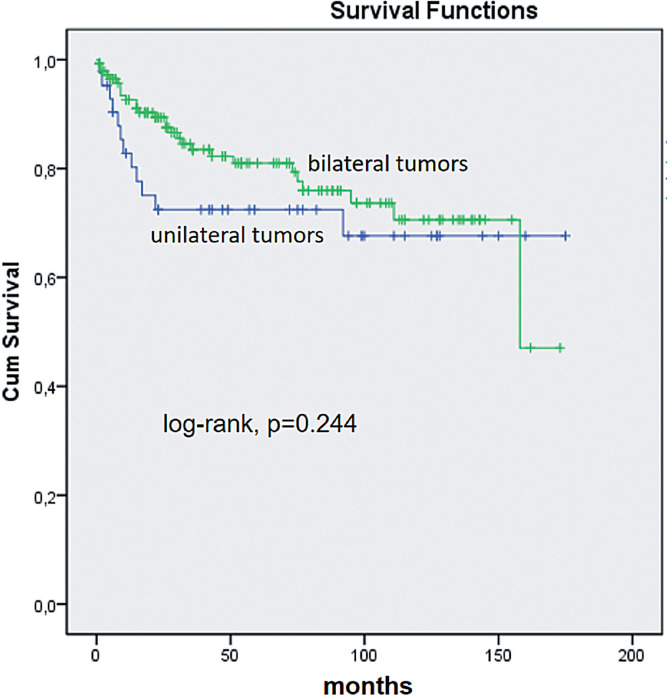
Kaplan - Meier product limit analysis shows time to PSA biochemical progression - free outcome by laterality considering total tumor extent. Cum, cumulative.

There was no significant different association with TBCR in Kaplan - Meier estimates. At 5 years of follow-up 69% of patients with unilateral tumors were BCR free vs. 83% with bilateral tumors (log rank p = 0.244, Figure-2) at a mean follow-up of 60 months (median 47, range 3 – 187).

### Risk of Shorter TBCR

On univariate Cox analysis, laterality did not significantly predict shorter time to PSA biochemical recurrence after surgery (HR 0.679, 95% CI 0.352 – 1.309, p = 248).

### Group 2 (index tumor extent)

In this group, 156 / 196 (79.6%) tumors were unilateral and 40 / 196 (20.4%) bilateral.

### Clinicopathological Findings

There was no significant association comparing bilateral vs. unilateral tumors (Table-2).

**Table 2:** Clinicopathological features of 156/196 (79.6%) unilateral tumors vs 40/196 (20.4%) bilateral tumors evaluated by index tumor extent.

Feature		Unilateral tumors	Bilateral tumors	p Value
Mean ± SD age/median (range)		63.05 ± 6.74/64.50 (46-76)	61.85 ± 5.35/62 (52-72)	0.162 (Mann-Whitney test)
Mean ± SD prostate weight/median (range)		42.74 ± 24.15/35.50 (15-185)	42.66 ± 22.76/38.50 (18-130)	0.822 (Mann-Whitney test)
**No. nodular hyperplasia (%)**				
	Neg	39 (25.3)	12 (30.0)	0.550 (Fisher exact test)
	Pos	115 (74.7)	28 (70.0)	
Mean ± SD preop PSA/median (range)		8.22 ± 5.33/6.96 (0.60-33)	6.82 ± 3.30/6.35 (0.60-14.60)	0.421 (Mann-Whitney test)
Mean ± SD PSA density/median (range)		0.21 ± 0.15/0.18 (0.03-1.10)	0.18 ± 0.12/0.16 (0.03-0.63)	0.145 (Mann-Whitney test)
**No. Grade group (%)**				
	1 (≤ 6)	79 (50.6)	19 (47.5)	0.461 (Qui-square test)
	2 (3+4=7)	63 (40.4)	17 (42.5)	
	3 (4+3=7)	12 (7.7)	2 (5.0)	
	4 (8)	0 (0.0)	1 (2.5)	
	5 (9-10)	2 (1.3)	1 (2.5)	
**No clinical stage (%)**				
	T1c	86 (57.3)	21 (56.8)	0.965 (Qui-square test)
	T2a/T2b	61 (40.7)		
	T2c	3 (2.0) 1 (2.7)	15 (40.5)	

### Pathological stage vs. clinical stage by DRE

Only 1 (2.7%) patient was considered cT2c by DRE vs. 40 (20.4%) patients pT2c; and, 15 (40.7%) patients were considered cT2a / cT2b by DRE vs. 156 (79.6%) patients pT2a / p2Tb.

### Time to BCR

**Figure 3:**
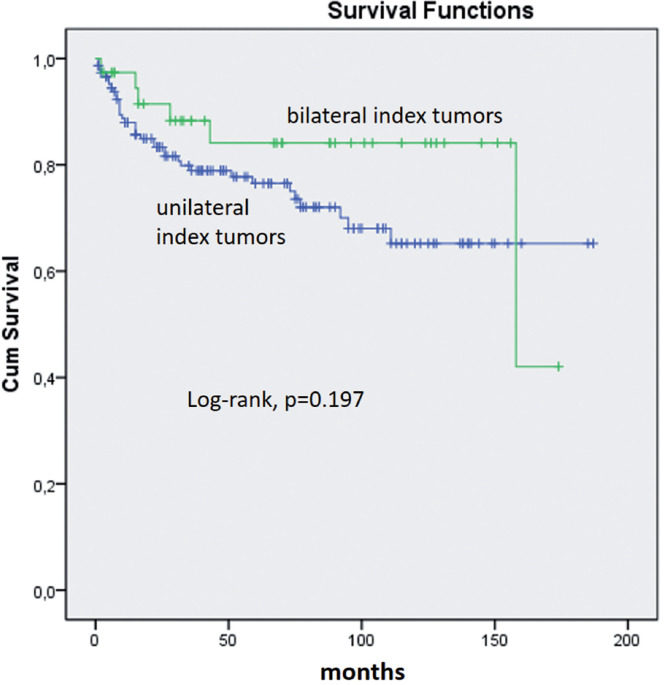
Kaplan - Meier product limit analysis shows time to PSA biochemical progression - free outcome by laterality considering index tumor extent. Cum, cumulative.

There was no significant association with TBCR in Kaplan - Meier estimates. At 5 years of follow-up 77% of patients with unilateral tumors were BCR free vs. 84% with bilateral tumors (log - rank, p = 0.197, Figure-3) at a mean follow-up of 60 months (median 47, range 3 – 187).

### Risk of Shorter TBCR

On univariate Cox regression analysis laterality did not significantly predict shorter time to BCR by index tumor extent (HR 0.571, 95% CI 0.240 – 1.357, p = 205).

## DISCUSSION

Our study validates the TNM 8^th^ edition for organ - confined prostate cancer. Pathologic sub - staging did not convey prognostic information either considering laterality as total tumor extent or index tumor extent. To the best of our knowledge, there is no other study considering index tumor extent in sub - staging. There were no significant clinicopathologic differences by laterality considering either total tumor extent or index tumor extent, and no significant difference for time to biochemical recurrence using Kaplan - Meier product limit analysis as well as prediction of shorter time using Cox stepwise logistic regression.

The multifocality seen in most prostate cancers is one major cause for absence of symmetry between clinical and pathological T2 sub - staging (6, 7) Prostate cancer may be extensive on one lobe (index tumor) and only insignificant on the other side.

There was no correlation between digital rectal examination and pathologic staging. The frequency of bilateral tumors (pT2c) evaluated by total tumor extent was 78.1% but only 2.8% were considered bilateral (cT2c); for unilateral tumors, 21.9% were pT2a / pT2b and 41.9% cT2a / cT2b. The frequency of bilateral tumors (pT2c) evaluated by index tumor extent was 20.4% but only 2.7% were considered bilateral (cT2c); for unilateral tumors, 79.6% were pT2a / pT2b and 40.7% cT2a / cT2b.

The objective of staging is: 1) to group malignancies with a similar prognosis and therapeutic approach; 2) to perform clinical trials or research studies on homogeneous patient populations; and, 3) to enhance the comparability of clinicopathologic data from hospitals and research groups across the World (8).

In general, pathologic staging (or sub - staging) tries to maintain symmetry with clinical staging (or sub - staging), allowing a direct comparison of both. The clinical staging of prostate cancer is a reflection of the detection methods employed and the sub - staging of clinical stage T2 prostate cancers is largely based on the extent of the abnormality palpated during a digital rectal examination or shown during transrectal ultrasonography in each half of the prostate (8).

The 1997 TNM staging system classified T2 prostate cancers into 2 groups: T2a (unilateral tumor) and T2b (bilateral tumor) (9). In 2002 and in 2009 the TNM staging system returned to the 1992 staging system classifying prostate cancers into 3 groups: T2a (unilateral tumor, involving less than half lobe), T2b (unilateral tumor, involving more than half lobe), and T2c (bilateral tumor) (10, 11).

During a consensus conference sponsored by the International Society of Urological Pathology (ISUP) on handling and staging of radical prostatectomy specimens held in Boston during the 98^th^ meeting of the United States and Canadian Academy of Pathology (USCAP), 65.5% of the attendants answered that the current pT2 sub - staging system should not be used (12). Answering to another question, 63.4% favored to be reduced to two categories based on studies showing that pathological T2b tumor does not exist (13-15).

Several studies have shown that pathologic T2 sub - staging does not convey prognostic information (13, 16-19). This paradox may be apparently explained in part by the fact that clinical criteria used in assessing stage indirectly estimate the chance of under - staging and in this way, they seem to stratify the heterogeneous group of clinical stage T2 patients (8). Smith and Catalona (20) found that the reproducibility of DRE for detecting prostate cancer is only fair among urologists. Probably, most palpable cT2b tumors are already pT2c or T3 disease, explaining why clinical staging has a better correlation with prognosis. Obek et al. (21) reviewed 89 patients with clinically palpable tumors (cT2) to assess whether the clinicians characterization of the disease as unilateral or bilateral by DRE correlated with the final pathology specimen. In 85 patients, a unilateral lesion was suspicious in DRE. The final pathological review revealed cancer on the suspicious side in 82 cases (96%) with tumor confined to the same lobe in only 23 (27%), bilateral disease in 59 (69%) and tumor confined to the contralateral lobe in 3 (4%). On the clinically benign side on DRE, there was a 36 and 31% incidence of extra - prostatic tumor extension and positive surgical margins, respectively.

A limitation of the current study is the small sample size and the relative short time of follow-up. The small sample may reflect the exclusion criteria in our series (stage pT2 and negative surgical margins). Local progression and distant metastases may develop even after 15 years of follow-up (22) however, more than 90% of patients experience recurrence within 5 years after surgery (23). Our point count method evaluates tumor extent but not volume and may not be accurate. Computer assisted analysis is the most precise method for tumor volume evaluation. The point count method ignores vertical tumor dimension but is equivalent to other methods that can be used by pathologists in routine practice (24, 25).

## CONCLUSIONS

The findings of the study validate the TNM 8^th^ edition for organ - confined prostate cancer. Pathologic sub - staging did not convey prognostic information either considering laterality as total tumor extent or index tumor extent. There were no significant clinicopathologic differences by laterality considering either total tumor extent or index tumor extent, and no significant difference for time to biochemical recurrence using Kaplan - Meier product limit analysis as well as prediction of shorter time using Cox stepwise logistic regression.

## ABBREVIATIONS

PSA: = Prostate specific antigen
RP: = Radical prostatectomy
BCR: = Biochemical recurrence
TBCR: = Time to BCR
DRE: = Digital rectal examination
